# Glycemic status and macronutrient intake as predictors of sleep outcomes: an analysis of NHANES 2007–2020 data

**DOI:** 10.3389/fnut.2025.1672631

**Published:** 2025-10-20

**Authors:** Raedeh Basiri, Megan Kassem, Yatisha Rajanala, Cara L. Frankenfeld

**Affiliations:** ^1^Department of Nutrition and Food Studies, George Mason University, Fairfax, VA, United States; ^2^Institute for Biohealth Innovation, George Mason University, Fairfax, VA, United States; ^3^Department of Global and Community Health, George Mason University, Fairfax, VA, United States; ^4^Department of Health Administration and Policy, George Mason University, Fairfax, VA, United States; ^5^Center for Interdisciplinary and Population Health Research, Maine Health Institute for Research, Westbrook, ME, United States

**Keywords:** diabetes, prediabetes, sleep disorders, sleep quality, sleep duration, glycemic status, macronutrient, NHANES

## Abstract

**Background:**

Emerging evidence suggests that glycemic status and dietary intake are associated with sleep duration and quality.

**Objective:**

To examine associations between glycemic status, diabetes control, macronutrient energy distribution, and sleep outcomes among participants of the National Health and Nutrition Examination Survey (NHANES).

**Methods:**

Sleep and dietary variables, including sleep duration (short/normal/extended), trouble sleeping, diagnosed sleep disorder, and macronutrient intake, were obtained from the cross-sectional US NHANES 2007–2020. Glycemic status was defined by self-reported diabetes history and measured HbA₁c. Multivariable adjusted multinomial logistic regression models were used to estimate odds ratios (ORs) and 95% confidence intervals (CIs) for sleep outcomes associated with glycemic status, diabetes control, and macronutrient intake.

**Results:**

Individuals with diabetes were more likely to have sleep disorders (OR: 1.61; 95% CI: 1.34–1.93) and trouble sleeping (OR: 1.37; 95% CI: 1.23–1.53) compared to those with normoglycemia. They also showed abnormal sleep durations, with 21% higher odds of short sleep (95% CI: 1.08–1.35) and 37% higher odds of extended sleep (95% CI: 1.12–1.66). Among participants with diabetes, maintaining HbA₁c < 6.5% was associated with 27% higher odds of trouble sleeping (95% CI: 1.05–1.54) versus those with 6.5% ≤ HbA₁c < 9.0%. Macronutrient distribution was differently associated with sleep across glycemic statuses. In individuals with diabetes, low protein intake was associated with higher odds of sleep disorder diagnosis (OR: 2.43; 95% CI: 1.06–5.61). A low-carbohydrate, high-fat intake was associated with lower odds of short sleep duration (OR: 0.78; 95% CI: 0.62–0.98). Among individuals with prediabetes, low-protein diets, particularly when combined with high fat intake, were associated with approximately 2- to 3-fold higher odds of extended sleep duration (OR: 2.04; 95% CI: 1.02–4.08; OR: 2.88; 95% CI: 1.30–6.36). In normoglycemic individuals, similar macronutrient energy distribution patterns were associated with both short and long sleep duration, compared with balanced diets.

**Conclusion:**

These findings highlight the importance of considering glycemic status and diet in relation to sleep. This study adds to growing evidence that metabolic health and nutrition influence sleep and may guide future interventions to improve sleep through targeted dietary strategies.

## 1 Introduction

Chronic sleep deprivation is a significant public health issue that affects millions of people worldwide and is defined as consistently getting less than the recommended 7–9 h of sleep per night (1, 2). Sleep disorders include a wide variety of conditions characterized by their ability to disrupt normal sleep patterns in both quantity and quality, causing severe physical, psychological, and sociological distress or disability (3). Sleep disorders contribute significantly to morbidity and mortality among U.S. adults, with approximately 35–40% reporting difficulty falling asleep or experiencing daytime sleepiness (4).

Prevalence data from the CDC’s Behavioral Risk Factor Surveillance System (2013–2022) highlights the persistent severity of insufficient sleep among U.S. adults, showing stable rates across both sexes over 9 years and underscoring the lack of progress in addressing the issue (5). The effects of sleep disorders on morbidity are closely associated with a number of anxiety disorders, mood disorders, depression, memory dysfunction, processing speed loss, executive function impairments, increased blood pressure and subsequent development of cardiovascular diseases, and increased risk of diabetes and obesity, due to its activation of cortisol release (3, 6, 7). Though the effects on mortality are relatively less known, it has been concluded that very short and extended sleep duration among men and women increases the relative risk of all-cause mortality, exceeding a 15% increased risk for both extended and short sleep durations (8, 9).

The economic implications of sleep disorders are also significant in the U.S., with the cost of “shift worker fatigue” estimated to result in a financial loss of billions of dollars (4). Based on data from the National Commission on Sleep Disorders Research (NCSDR), insomnia alone accounts for a $13.9 billion loss in direct costs to the country, with additional indirect costs related to absenteeism and reduced productivity (10). Pharmacological treatments for sleep disorders include melatonin receptor agonists, orexin receptor agonists, non-benzodiazepine hypnotics, histamine type-1 receptor blockers, and benzodiazepines. However, these treatments are highly subject to tolerance and can lead to dependence (11, 12). As such, identifying risk factors and enhancing prevention efforts are critical to reducing the burden of sleep disorders and their associated comorbidities.

Research has shown a strong association between sleep deprivation and type 2 diabetes (13–17). Sleep deprivation can lead to insulin resistance, impairing the body’s ability to regulate blood glucose effectively (16, 18, 19). A study by Arora and Taheri highlighted that individuals with short sleep duration of less than or equal to 5 h per night had 251% higher odds of developing type 2 diabetes compared to those who 7-8 h per night (20). Not only does impaired sleep increase the risk of developing type 2 diabetes, but having diabetes itself can also negatively impact both sleep quality and duration (21).

On the other hand, nutrition plays a crucial role in managing sleep quality and overall health in individuals with prediabetes or diabetes (22–30). A cohort study by Nôga et al., involving 247,867 participants, found that extreme short sleep duration (3–4 h/day) at baseline was associated with a 41% higher risk of developing type 2 diabetes over an average of a 12-year follow-up period. This study indicated that individuals who adhered to the healthiest diet had a 25% reduction in the risk of developing type 2 diabetes (31).

Macronutrients, including carbohydrates, proteins, and fats, have specific impacts on sleep, mental and metabolic health (32–35). Carbohydrates can influence sleep quality by affecting the production of serotonin and melatonin, neurotransmitters that regulate sleep (36, 37). Consuming complex carbohydrates, such as whole grains, can promote better sleep by stabilizing blood sugar levels (38), whereas greater consumption of low-quality carbohydrates and total daily carbohydrate intake is linked to a 39% and 31% higher risk of poor sleep habits, respectively (39). Proteins are essential to produce amino acids, which are precursors to neurotransmitters like serotonin and melatonin (36). A diet rich in lean proteins, such as fish, poultry, and legumes, can support healthy sleep patterns (40). High-fat diets have been correlated with reduced sleep efficiency and rapid-eye movement sleep. Saturated fats, in particular, have been associated with shortened duration of slow-wave sleep and more frequent arousals, factors that result in diminished sleep quality (37). Studies have also shown that individuals with insomnia tend to consume more high-fat foods compared to those without sleep disorders (41, 42).

Poor sleep, inadequate glycemic control, and suboptimal macronutrient composition create a vicious cycle that can further degrade both metabolic health and sleep quality. Investigating the interplay of glycemic status, diet, and sleep across normoglycemia, prediabetes, and diabetes is critical, as sleep disturbances increase with worsening glucose intolerance and further impair glycemic control (43–45). This study examined associations between glycemic status, diabetes control, macronutrient energy distribution, and sleep outcomes across different glycemic groups among U. S. adults using the National Health and Nutrition Examination Surveys (NHANES) data. The specific objectives of this study are outlined below:

1. To compare sleep duration categories (short, normal, extended) and sleep quality (trouble sleeping, diagnosed sleep disorder) across three glycemic-status groups (normoglycemia, prediabetes, and diabetes) in U. S. adults using NHANES 2007–2020 data.
2. To evaluate the association between diabetes control, defined by HbA₁c thresholds (HbA₁c < 6.5%, 6.5% ≤ HbA₁c < 9.0%, HbA₁c ≥ 9.0%), and sleep outcomes among participants with diabetes.
3. To assess how different macronutrient energy distribution patterns relate to sleep duration and quality within each glycemic-status group.

Clarifying these relationships can inform integrated diet–sleep interventions aimed at optimizing HbA₁c targets, preventing diabetes progression, and enhancing overall disease management.

## 2 Materials and methods

### 2.1 Source of data

Data from the National Health and Nutrition Examination Surveys (NHANES) was used for this study. NHANES is a series of cross-sectional surveys of a nationally representative sample that has been conducted continuously since 1999 and released in two-year data cycles (46). The Centers for Disease Control and Prevention (CDC) uses NHANES to assess the health and nutritional status of the U.S. non-institutionalized population through interviews, laboratory tests, physical examinations, and dietary recalls. A complex and multistage probability-sampling design is used to maintain national representativeness by segregating the country into Primary Sampling Units (PSUs) based on region and urbanization (47).

For this study, data was extracted on 66,148 participants who participated in NHANES from 2007 to 2020. Participants included in the analysis were non-pregnant, non-lactating adults aged 18 years or older with available data on hemoglobin A1c (HbA₁c) values/diabetes diagnosis. For the sub-analyses relating to macronutrient consumption, only participants who had reliable dietary recalls and had total daily energy intake between 500 and 3,500 kcals (for females) and 800 and 4,200 kcals (for males) were included (48). Dietary records were determined to be reliable by NHANES data collectors and was based on whether or not they met all three of the following criteria: (1) No more than 25% of reported food items were missing descriptive information (e.g., caffeinated or decaffeinated, preparation methods, or brand names); (2) No more than 15% of reported foods were missing amounts; and, (3) Any reported meal must include at least one known food [i.e., if a participant reported having lunch on the recalled day but was unable to recall or report any specific foods from that meal, the recall did not meet the criterion (49)].

**Figure 1:**
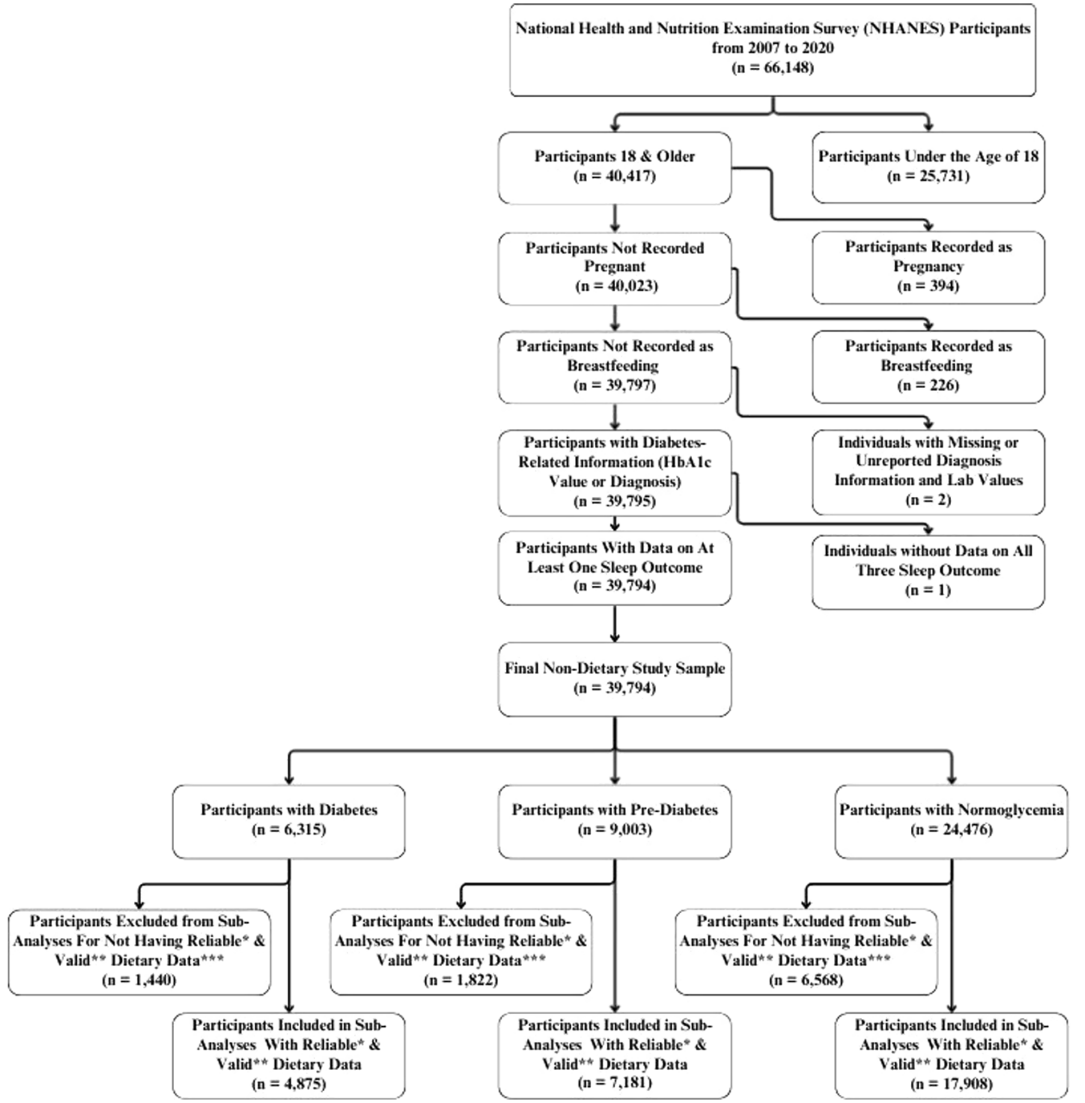
Flowchart of eligible individuals for the study from the National Health and Nutrition Examination Survey (NHANES) 2007–2020. *Recall records were determined by NHANES data collectors to be reliable if they met the following minimum criteria: • No more than 25% of reported food items were missing descriptive information (e.g., caffeinated or decaffeinated, preparation methods, or brand names). • No more than 15% of reported foods were missing amounts. • Any reported meal must include at least one known food [i.e. if a participant reported having lunch on the recalled day but was unable to recall or report any specific foods from that meal, the recall did not meet the criterion (49)]. **Valid records were defined as those with total energy intakes between 500–3500 kcal/day for women and 800–4200 kcal/day for men (48). ***Because the categories of over-consumed protein, over-consumed carbohydrates, and under-consumed fats represented a very small proportion of participants, they were excluded from the analysis due to concerns regarding statistical power and the reliability of estimates.

Furthermore, among the selected participants, those who were missing a response, refused to provide a response, or had reported being unsure of their sleep quality/quantity were excluded, from their respective subanalyses. For instance, participants who had sleep quality data without sleep duration were excluded from the analyses of sleep duration but included within the population for troubled sleeping. Based on these criteria, the final analysis included a total of 39,794 participants, as depicted in Figure 1. This group constituted the subpopulation used in statistical analyses to maintain appropriate population weights.

### 2.2 Sleep quality

The participants’ sleep quality was assessed based on their responses to the 2007–2020 question (“{Have you/Has SP} ever told a doctor or other health professional that {you have/s/he has} trouble sleeping?”) and the 2007–2014 question (“{Have you/Has SP} ever been told by a doctor or other health professional that {you have/s/he has} a sleep disorder?”). The sleep disorder diagnosis question was discontinued after the 2013–2014 cycle. From 2015 to 2020, only self-reported trouble sleeping was available and used as a qualitative measure of sleep health. Sleep duration was determined by responses to the questions of (“How much sleep {do you/does SP} usually get at night on weekdays or workdays?” (2007–2014) and “Number of hours usually sleep on weekdays or workdays.” (2017–2020). From the reported hours of sleep, participants were classified into three categories: short (sleep duration < 7 h), normal (7 h ≤ sleep duration ≤ 9 h), and extended (sleep duration > 9 h) (50–52).

### 2.3 Diabetes status

The participants’ diabetes status was determined based on prior diagnosis by a provider and/or by the level of HbA₁c from laboratory examinations (53). Those who had a previous diagnosis of diabetes were additionally classified based on their HbA₁c levels as diabetes with HbA₁c < 6.5%, diabetes with 6.5% ≤ HbA₁c < 9.0%, and diabetes with HbA₁c ≥ 9.0%. Participants who reported no prior diagnosis of diabetes based on responses to question “Other than during pregnancy, have you ever been told by a doctor or health professional that you have diabetes or sugar diabetes?” were classified by HbA₁c levels as follows: normoglycemia (HbA₁c < 5.7%), prediabetes (5.7% ≤ HbA₁c < 6.5%), or diabetes (HbA₁c ≥ 6.5%). These participants were then merged with those who self-reported a diabetes diagnosis.

### 2.4 Macronutrient distribution

Macronutrient intake from the dietary recall questionnaires was classified according to the Acceptable Macronutrient Distribution Ranges (AMDR) guidelines (54). Intake was considered within the recommended range if carbohydrates comprised 45–65% of total energy, protein 10–35%, and fat 20–35%. Values outside these ranges were classified as underconsumption or overconsumption (54, 55). To determine this, data from the first-day dietary interview, specifically total nutrient intake from food recall questionnaires collected through in-person interviews, were extracted. Each macronutrient (present in grams) was converted to calories, from which the percent calorie intake was calculated from the total energy intake. Calories from alcohol were included in total energy intake but excluded from macronutrient distribution patterns, as alcohol is not defined as a macronutrient and no AMDR values exist for it. Eligible participants who over-consumed protein (0.65%), over-consumed carbohydrates (4.30%), or under-consumed fat (5.51%) were few and thus, were excluded from the analyses. We defined eight macronutrient energy distribution patterns in this population based on whether participants met the AMDR thresholds for protein, carbohydrates, and fat. The low-protein, low-carbohydrate pattern (protein <10%, carbohydrate <45%, 20% ≤ fat ≤ 35%) had a very low prevalence (Supplementary Table 1), which resulted in limited statistical power and unstable estimates. Therefore, results for this group are not reported, and all analyses were reported using the seven remaining macronutrient energy distribution patterns, as outlined below:

Balanced Diet: 10% ≤ Protein ≤ 35%, 45% ≤ Carbohydrate ≤ 65%, 20% ≤ Fat ≤ 35%.
Low-Protein Diet: Protein <10%, 45% ≤ Carbohydrate ≤ 65%, 20% ≤ Fat ≤ 35%.
Low-Protein, High-Fat Diet: Protein <10%, 45% ≤ Carbohydrate ≤ 65%, Fat >35%.
High-Fat Diet: 10% ≤ Protein ≤ 35%, 45% ≤ Carbohydrate ≤ 65%, Fat >35%.
Low-Carbohydrate, High-Fat Diet: 10% ≤ Protein ≤ 35%, Carbohydrate <45%, Fat >35%.
Low-Carbohydrate Diet: 10% ≤ Protein ≤ 35%, Carbohydrate <45%, 20% ≤ Fat ≤ 35%.
Unbalanced Diet: Protein <10%, Carbohydrate <45%, Fat >35%.

### 2.5 Demographic, anthropometric, and physical activity data

Age, gender, survey year, race/ethnicity, physical activity, and body mass index (BMI) were included as covariates. Weight and height were measured by trained technicians during the in-person interview and used to calculate BMI. Using this information, individuals were classified into six BMI groups: underweight (BMI < 18.5 kg/m^2^), normal weight (18.5 kg/m^2^ ≤ BMI < 25 kg/m^2^), overweight (25 kg/m^2^ ≤ BMI < 30 kg/m^2^), class I obese (30 kg/m^2^ ≤ BMI < 35 kg/m^2^), class II obese (35 kg/m^2^ ≤ BMI < 40 kg/m^2^), and class III obese (BMI ≥ 40 kg/m^2^). Participants with no BMI information were assigned to a missing category. Physical activity levels were assessed based on the activity reported by the individuals through the Global Physical Activity Questionnaire (GPAQ), which was used to calculate Metabolic Equivalent of Task (MET) hours per week (MET-hrs./week) (53, 56).

### 2.6 Statistical analysis

To assess sleep outcomes, we used SAS 9.4 to perform multinomial logistic regression and estimate the odds of sleep disorder diagnosis, trouble sleeping, and abnormal sleep duration across three domains: (1) Glycemic Status, comparing participants with prediabetes and diabetes to those with normoglycemia; (2) Diabetes Control, comparing individuals with diabetes and HbA₁c < 6.5% or ≥ 9.0% to those with 6.5% ≤ HbA₁c < 9.0%; and (3) Macronutrient Distribution, comparing those adhering to a balanced diet against all other dietary patterns. Analyses were conducted using three models: an unadjusted model; a semi-adjusted model controlling for age, sex, and race/ethnicity; and a fully adjusted model that additionally included survey year, body mass index (BMI), and physical activity. Using three models allowed us to evaluate the robustness of associations and understand the influence of potential confounders. The unadjusted model shows the raw association, the semi-adjusted model accounts for key demographic factors, and the fully adjusted model further controls for lifestyle and temporal variables. For all models, the statistical significance *p*-value was set to <0.05.

The analyses applied NHANES-provided sampling weights to account for the complex survey design, including oversampling, non-response (across the interview, mobile examination center [MEC], and dietary assessment stages), and stratification, ensuring estimates are representative of the U.S. population. For analyses involving combined survey years, multi-year interview weights were constructed: MEC weights were used for analyses of diabetes status and glycemic control, and Day 1 dietary sample weights were used for analyses of macronutrient intake (57).

## 3 Results

The demographic and sleep characteristics of the study population, stratified by glycemic status, are reported in Table 1. In summary, 38.49% of the population had either prediabetes or diabetes. Among participants with diabetes, males represented a higher proportion (51.84%) than females (48.16%). Individuals with diabetes had the highest prevalence of trouble sleeping (37.74%) and diagnosed sleep disorders (9.56%), compared to those with prediabetes (30.88% and 5.89%, respectively) and normoglycemia (24.91% and 3.95%, respectively). Short sleep (<7 h) was also more common among participants with diabetes (33.59%) and prediabetes (31.29%) than those with normoglycemia (29.57%). Additionally, extended sleep (>9 h) was more common among individuals with diabetes (7.79%) than those with prediabetes (5.35%) or normoglycemia (5.24%). The distribution of macronutrient energy distribution patterns indicated that the low-carbohydrate, high-fat diet was the most common across all glycemic status groups, followed by the balanced diet. Overall, 38.65% of participants adhered to a low-carbohydrate, high-fat diet, and 31.38% followed a balanced diet. Among those with diabetes, nearly half (46.46%) were classified as consuming a low-carbohydrate, high-fat diet, while only 27.56% were characterized by a balanced diet. Similar trends were observed among participants with normoglycemia and prediabetes. Unbalanced diets accounted for substantially smaller proportions of participants. More details about the distribution of macronutrient consumption patterns stratified by glycemic status are provided in Supplementary Table 1.

**Table 1:** Demographic and sleep characteristics of NHANES participants eligible for the study (2007–2020), stratified by glycemic status.

Participant Characteristics	Total participants (*n* = 39,794)	Participants with normoglycemia (*n* = 24,476)	Participants with prediabetes (*n* = 9,003)	Participants with diabetes (*n* = 6,315)
Age				
18–34 Years	29.14% (*n* = 10,996)	39.22% (*n* = 9,768)	10.50% (*n* = 993)	4.45% (*n* = 235)
35–44 Years	17.17% (*n* = 6,272)	19.53% (*n* = 4,580)	13.75% (*n* = 1,173)	9.79% (*n* = 519)
45–54 Years	18.28% (*n* = 6,386)	17.27% (*n* = 3,628)	20.59% (*n* = 1,697)	20.04% (*n* = 1,061)
55–64 Years	16.69% (*n* = 6,717)	12.73% (*n* = 2,904)	24.07% (*n* = 2,126)	26.25% (*n* = 1,687)
65 + Years	18.72% (*n* = 9,423)	11.25% (*n* = 3,596)	31.08% (*n* = 3,014)	39.48% (*n* = 2,813)
Sex				
Male	49.06% (*n* = 19,658)	49.11% (*n* = 11,985)	47.26% (*n* = 4,426)	51.84% (*n* = 3,247)
Female	50.94% (*n* = 20,136)	50.89% (*n* = 12,491)	52.74% (*n* = 4,577)	48.16% (*n* = 3,068)
Race/Ethnicity				
Non-Hispanic White	65.45% (*n* = 15,706)	68.04% (*n* = 10,466)	61.24% (*n* = 3,220)	58.13% (*n* = 2,020)
Non-Hispanic Black	11.45% (*n* = 8,981)	9.43% (*n* = 4,693)	15.60% (*n* = 2,523)	15.66% (*n* = 1,765)
Mexican American	8.61% (*n* = 5,828)	8.40% (*n* = 3,497)	8.47% (*n* = 1,254)	9.99% (*n* = 1,077)
Non-Mexican Hispanic	6.22% (*n* = 4,251)	6.28% (*n* = 2,599)	5.94% (*n* = 943)	6.36% (*n* = 709)
Other Races-Including Multi-Racial	8.28% (*n* = 5,028)	7.85% (*n* = 3,221)	8.76% (*n* = 1,063)	9.86% (*n* = 744)
BMI categories				
Underweight	1.68% (*n* = 681)	2.11% (*n* = 563)	1.07% (*n* = 94)	0.33% (*n* = 24)
Normal weight	28.05% (*n* = 10,416)	33.94% (*n* = 7,911)	19.02% (*n* = 1,769)	10.41% (*n* = 736)
Overweight	32.10% (*n* = 12,054)	33.03% (*n* = 7,379)	33.31% (*n* = 3,013)	24.77% (*n* = 1,662)
Class I Obese	20.41% (*n* = 7,759)	18.06% (*n* = 3,996)	23.23% (*n* = 2,100)	28.78% (*n* = 1,663)
Class II Obese	9.39% (*n* = 3,641)	7.01% (*n* = 1,624)	12.79% (*n* = 1,060)	16.93% (*n* = 957)
Class III Obese	7.16% (*n* = 2,825)	4.75% (*n* = 1,098)	9.59% (*n* = 834)	16.57% (*n* = 893)
Missing	1.21% (*n* = 2,418)	1.10% (*n* = 1,905)	0.99% (*n* = 133)	2.21% (*n* = 380)
Physical activity				
No physical activity	22.25% (*n* = 10,706)	18.05% (*n* = 5,424)	26.62% (*n* = 2,667)	38.36% (*n* = 2,615)
Quartile 1	18.90% (*n* = 7,638)	17.93% (*n* = 4,442)	21.46% (*n* = 1,868)	19.98% (*n* = 1,328)
Quartile 2	21.00% (*n* = 7,512)	22.22% (*n* = 4,925)	18.47% (*n* = 1,582)	18.45% (*n* = 1,005)
Quartile 3	18.08% (*n* = 6,508)	20.01% (*n* = 4,467)	15.57% (*n* = 1,341)	11.50% (*n* = 700)
Quartile 4	19.77% (*n* = 7,424)	21.78% (*n* = 5,212)	17.88% (*n* = 1,545)	11.72% (*n* = 667)
Missing	0.01% (*n* = 6)	0.01% (*n* = 6)	0.00% (*n* = 0)	0.00% (*n* = 0)
Trouble Sleeping				
Trouble sleeping	27.68% (*n* = 10,066)	24.91% (*n* = 5,445)	30.88% (*n* = 2,409)	37.74% (*n* = 2,212)
No trouble sleeping	72.29% (*n* = 29,707)	75.06% (*n* = 19,016)	69.10% (*n* = 6,591)	62.23% (*n* = 4,100)
Missing	0.02% (*n* = 21)	0.03% (*n* = 15)	0.02% (*n* = 3)	0.03% (*n* = 3)
Sleep disorder				
Disordered sleeping	5.02% (*n* = 1,982)	3.95% (*n* = 931)	5.89% (*n* = 500)	9.56% (*n* = 551)
No disordered sleeping	53.94% (*n* = 22,341)	56.08% (*n* = 14,350)	52.31% (*n* = 4,932)	44.77% (*n* = 3,059)
Missing	41.03% (*n* = 15,471)	39.97% (*n* = 9,195)	41.80% (*n* = 3,571)	45.67% (*n* = 2,705)
Sleep duration				
Short sleep duration (<7 Hours)	30.41% (*n* = 13,101)	29.57% (*n* = 7,735)	31.29% (*n* = 3,140)	33.59% (*n* = 2,226)
Normal sleep duration (7–9 Hours)	63.69% (*n* = 23,866)	64.93% (*n* = 15,109)	63.02% (*n* = 5,281)	57.90% (*n* = 3,476)
Extended sleep duration (> 9 Hours)	5.57% (*n* = 2,664)	5.24% (*n* = 1,549)	5.35% (*n* = 546)	7.79% (*n* = 569)
Missing	0.33% (*n* = 163)	0.25% (*n* = 83)	0.34% (*n* = 36)	0.72% (*n* = 44)

Proportions (%) are weighted using NHANES sampling weights to represent the noninstitutionalized resident US population. Counts (n) are unweighted and represent the number of study participants within each subgroup.

### 3.1 Glycemic status and sleep outcomes across glycemic categories

In the fully adjusted model, diabetes was strongly associated with multiple adverse sleep outcomes, while pre-diabetes showed limited associations. Compared with normoglycemic participants, individuals with diabetes had higher odds of reporting trouble sleeping (OR = 1.37; 95% CI: 1.23–1.53), a sleep disorder diagnosis (OR = 1.61; 95% CI: 1.34–1.93), short sleep duration (OR = 1.21; 95% CI: 1.08–1.35), and extended sleep duration (OR = 1.37; 95% CI: 1.12–1.66). In contrast, pre-diabetes was only modestly associated with trouble sleeping in the fully-adjusted model (OR = 1.09; 95% CI: 1.00–1.18). Detailed odds ratios and 95% confidence intervals for the associations between glycemic status and sleep outcomes are presented in Table 2. When adjusting only for gender, race/ethnicity, and age, sleep disorder diagnosis (OR = 1.42; 95% CI: 1.19–1.68), trouble sleeping (OR = 1.19; 95% CI: 1.09–1.29), and short sleep duration (OR = 1.08; 95% CI: 1.01–1.16) were also significantly associated with prediabetes (Supplementary Table 2). However, across all models, the findings indicate that sleep disturbances are most consistently elevated in individuals with diabetes rather than pre-diabetes.

**Table 2:** Odds ratios and 95% confidence intervals for associations between glycemic status and sleep outcomes among NHANES participants (*n* = 39,794).

Sleep Characteristics	Fully adjusted model^1^		
	OR	Lower limit	Upper limit
Sleep quality			
Trouble sleeping			
Normoglycemia	1.00	Ref	Ref
Prediabetes	1.09	1.00	1.18
Diabetes	1.37	1.23	1.53
Sleep disorder diagnosis			
Normoglycemia	1.00	Ref	Ref
Prediabetes	1.15	0.97	1.35
Diabetes	1.61	1.34	1.93
Sleep quantity			
Short (<7 h) duration			
Normoglycemia	1.00	Ref	Ref
Prediabetes	1.03	0.96	1.11
Diabetes	1.21	1.08	1.35
Extended (>9 h) duration			
Normoglycemia	1.00	Ref	Ref
Prediabetes	0.99	0.86	1.14
Diabetes	1.37	1.12	1.66

1 Depicts the results of a full-adjusted model, adjusting for sex (men and women), age (18–34, 35–44, 45–54, 55–64, and ≥65 years), race/ethnicity (Non-Hispanic White, Mexican American, Non-Mexican Hispanic, Non-Hispanic Black, and other races—including multi-racial), BMI [underweight (<18.5 kg/m^2^), normal weight (≥18.5 and <25 kg/m^2^), overweight (≥25 and <30 kg/m^2^), class I obese (≥30 and <35 kg/m^22^), class II obese (≥35 and <40 kg/m^2^), class III obese (≥40 kg/m^2^), and missing values], year of collecting data (continuous), and physical activity (MET-hrs./week = 0, quartiles, missing).

### 3.2 Diabetes control status and sleep outcomes in adults with diabetes

Compared to those with 6.5% ≤ HbA₁c < 9.0%, the odds of having trouble sleeping were 27% (95% CI: 1.05–1.54) higher among individuals with HbA₁c < 6.5% in the fully adjusted model as presented in Table 3. In the unadjusted model, individuals with HbA₁c ≥ 9 had 35% higher odds of short sleep duration (95% CI: 1.03–1.77); however, this association was no longer statistically significant after adjusting for demographic and lifestyle factors (Supplementary Table 3).

**Table 3:** Odds ratios and 95% confidence intervals for associations between diabetes control status and sleep outcomes among participants in NHANES (*n* = 5,855).

Sleep Characteristics	Fully adjusted model^1^		
	OR	Lower limit	Upper limit
Sleep quality			
Trouble sleeping			
Diabetes (HbA₁c ≥ 9)	0.99	0.79	1.24
Diabetes (6.5 ≤ HbA₁c < 9)	1.00	Ref	Ref
Diabetes (HbA₁c < 6.5)	1.27	1.05	1.54
Sleep disorder diagnosis			
Diabetes (HbA₁c ≥ 9)	0.97	0.61	1.55
Diabetes (6.5 ≤ HbA₁c < 9)	1.00	Ref	Ref
Diabetes (HbA₁c < 6.5)	1.32	0.91	1.91
Sleep quantity			
Short (<7 h) duration			
Diabetes (HbA₁c ≥ 9)	1.21	0.91	1.60
Diabetes (6.5 ≤ HbA₁c < 9)	1.00	Ref	Ref
Diabetes (HbA₁c < 6.5)	1.02	0.85	1.22
Extended (>9 h) duration			
Diabetes (HbA₁c ≥ 9)	0.83	0.54	1.29
Diabetes (6.5 ≤ HbA₁c < 9)	1.00	Ref	Ref
Diabetes (HbA₁c < 6.5)	1.01	0.73	1.40

1 Depicts the results of a full-adjusted model, adjusting for sex (men and women), age (18–34, 35–44, 45–54, 55–64, and ≥65 years), race/ethnicity (Non-Hispanic White, Mexican American, Non-Mexican Hispanic, Non-Hispanic Black, and other races—including multi-racial), BMI [underweight (<18.5 kg/m^2^), normal weight (≥18.5 and <25 kg/m^2^), overweight (≥25 and <30 kg/m^2^), class I obese (≥30 and <35 kg/m^2^), class II obese (≥35 and <40 kg/m^2^), class III obese (≥40 kg/m^2^), and missing values], year of collecting data (continuous), and physical activity (MET-hrs./week = 0, quartiles, missing).

### 3.3 Macronutrient intake and sleep outcomes across glycemic categories

Several macronutrient energy distribution patterns were significantly associated with sleep outcomes, with notable variation across glycemic status groups. Consistently across groups, low-protein diets were linked to multiple adverse sleep outcomes. Among participants with diabetes, a low-protein diet was associated with more than a 2-fold increase in the odds of having a diagnosed sleep disorder (OR = 2.43; 95% CI: 1.06–5.61). In individuals with prediabetes, low-protein intake was associated with more than double the odds of extended sleep duration (OR = 2.04; 95% CI: 1.02–4.08). Among those with normoglycemia, low-protein intake was linked to 31% higher odds of short sleep duration (95% CI: 1.01–1.71) and 72% higher odds of extended sleep duration (95% CI: 1.05–2.80).

Low-protein, high-fat diets demonstrated similarly unfavorable associations. In participants with diabetes, no significant associations were observed. However, in those with prediabetes, this dietary pattern was associated with nearly a 3-fold increase in extended sleep duration (OR = 2.88; 95% CI: 1.30–6.36). Among normoglycemic individuals, low-protein, high-fat diets were associated with 49% higher odds of short sleep duration (95% CI: 1.07–2.06) and more than double the odds of extended sleep duration (OR = 2.10; 95% CI: 1.30–3.40).

By contrast, low-carbohydrate, high-fat diets were associated with more favorable outcomes. In participants with diabetes, this pattern was linked to a 22% reduction in the odds of short sleep duration (OR = 0.78; 95% CI: 0.62–0.98). No significant associations were observed in those with prediabetes. Among individuals with normoglycemia, low-carbohydrate, high-fat diets were associated with a 15% reduction in the odds of short sleep duration (OR = 0.85; 95% CI: 0.76–0.96).

Other dietary patterns, including high-fat, unbalanced, and low-carbohydrate diets, were not significantly associated with sleep outcomes across glycemic status groups in fully adjusted models. However, in models adjusted only for non-modifiable demographic characteristics, high-fat diets were associated with a 77% increase in the odds of extended sleep duration among individuals with prediabetes (95% CI: 1.02–3.06; Supplementary Table 5), and low-carbohydrate diets were associated with 52% lower odds of extended sleep duration in individuals with diabetes (95% CI: 0.24–0.96; Supplementary Table 5).

**Figure 2:**
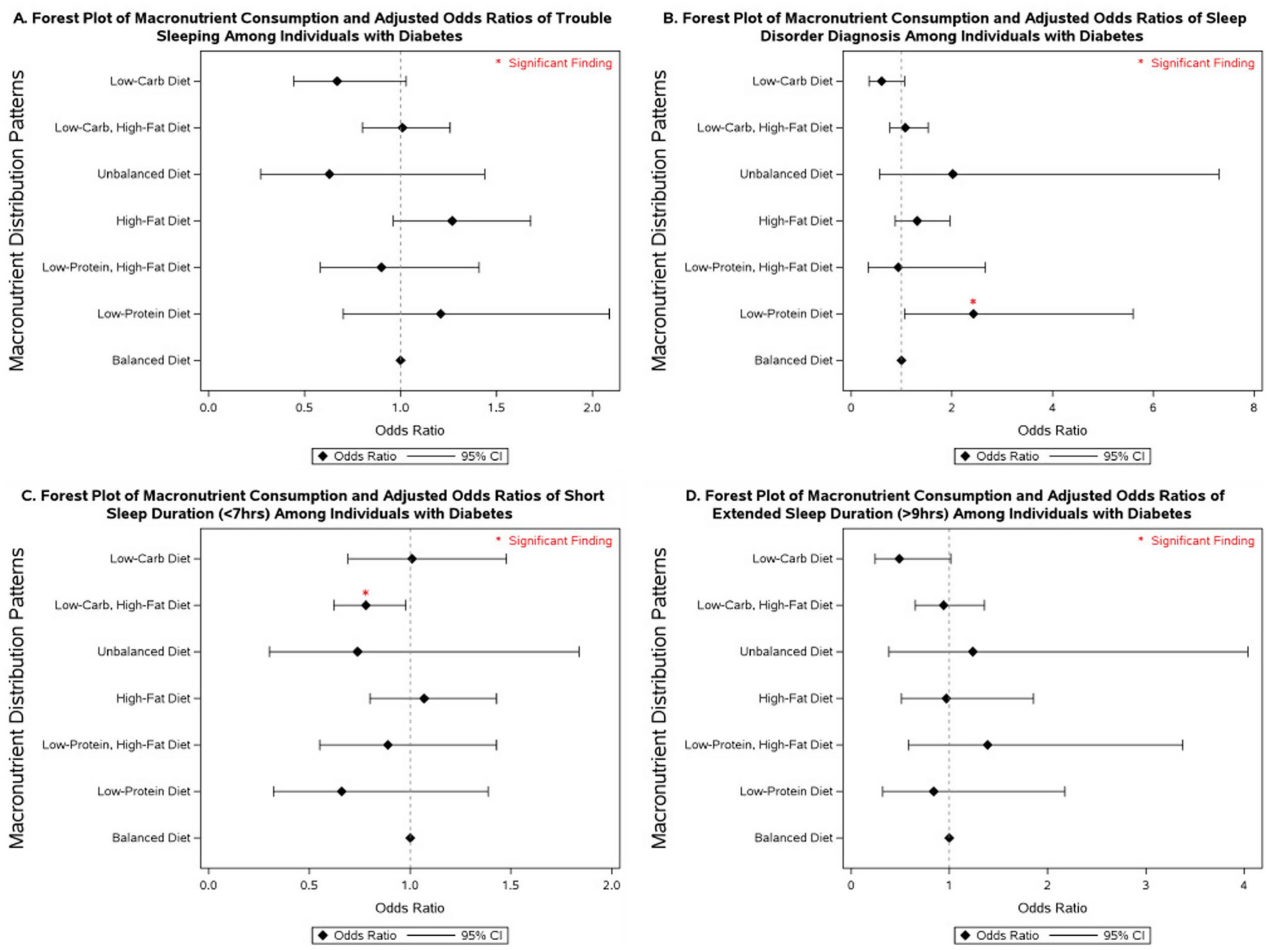
Forest plot of fully adjusted^1^ odds ratios for the associations between macronutrient intake^2^ and sleep outcomes among participants with diabetes in NHANES 2007–2020 (*n* = 4,875). Black diamonds represent odds ratios (ORs), with horizontal lines indicating 95% confidence intervals (CIs); statistically significant findings are marked with a red asterisk. ^1^The model adjusts for sex (men and women), age (18–34, 35-44, 45-54, 55–64, and ≥65 years), race/ethnicity (Non-Hispanic White, Mexican American, Non-Mexican Hispanic, Non-Hispanic Black, and other races including multi-racial), BMI [underweight (<18.5 kg/m^2^), normal weight (≥18.5 and <25 kg/m^2^), overweight (≥25 and <30 kg/m^2^), class I obese (≥30 and <35 kg/m^2^), class II obese (≥35 and <40 kg/m^2^), class III obese (≥40 kg/m^2^), and missing values], year of collecting data, and physical activity (MET-hrs./week = 0, quartiles, missing). ^2^Diets were classified based on macronutrient energy distribution. A balanced diet was defined as 10–35% protein, 45–65% carbohydrate, and 20–35% fat. Low-protein: protein <10%, carbohydrate 45–65%, fat 20–35%. Low-protein, high-fat: protein <10%, carbohydrate 45–65%, fat >35%. High-fat: protein 10–35%, carbohydrate 45–65%, fat >35%. Unbalanced (all off-range): protein <10%, carbohydrate <45%, fat >35%. Low-carbohydrate, high-fat: protein 10–35%, carbohydrate <45%, fat >35%. Low-carbohydrate: protein 10–35%, carbohydrate <45%, fat 20–35%.

**Figure 3:**
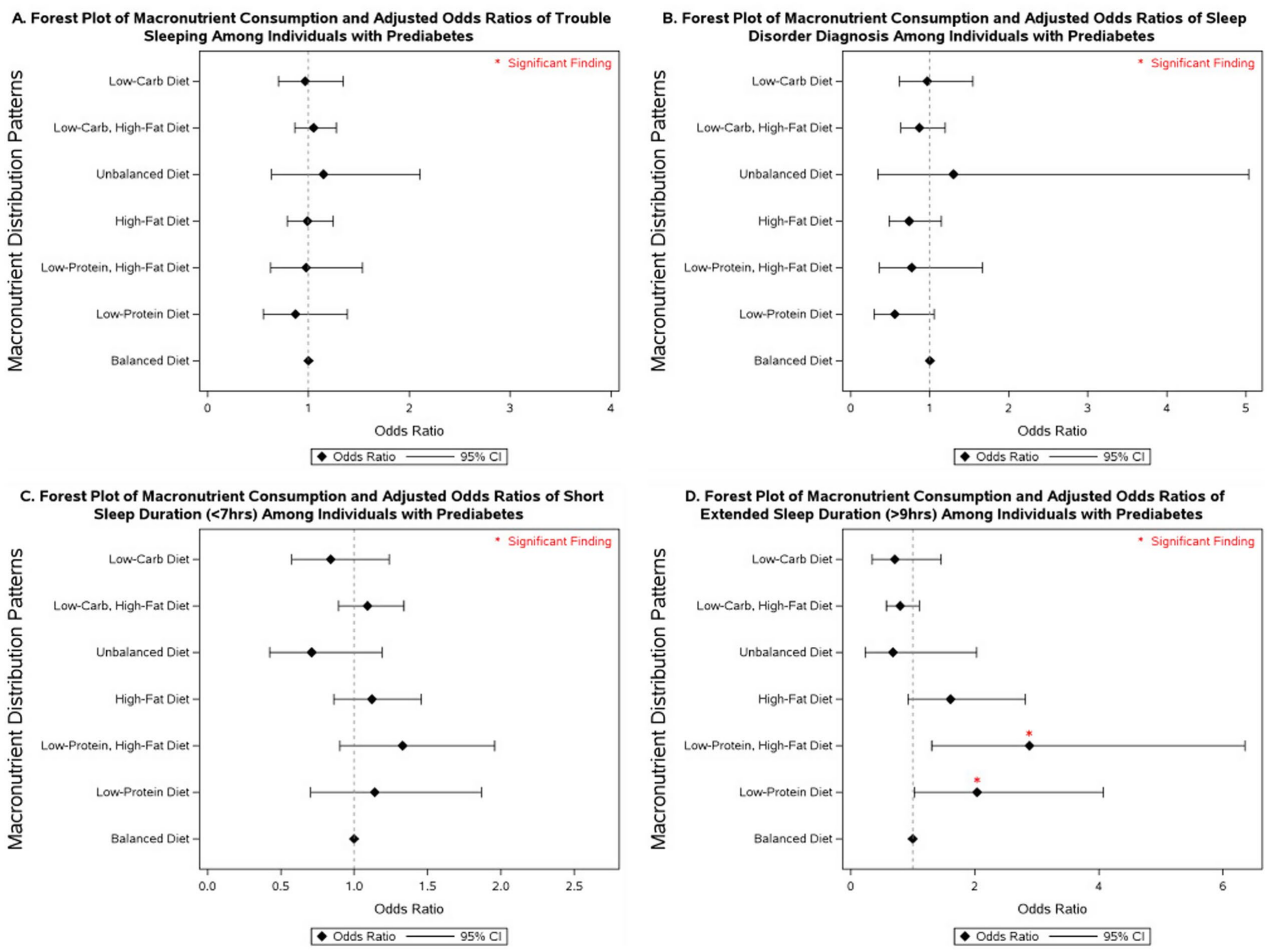
Forest plot of fully adjusted^1^ odds ratios for the associations between macronutrient intake^2^ and sleep outcomes among participants with diabetes in NHANES 2007–2020 (*n* = 7,181). Black diamonds represent odds ratios (ORs), with horizontal lines indicating 95% confidence intervals (CIs); statistically significant findings are marked with a red asterisk. ^1^The model adjusts for sex (men and women), age (18-34, 35-44, 45-54, 55–64, and ≥65 years), race/ethnicity (Non-Hispanic White, Mexican American, Non-Mexican Hispanic, Non-Hispanic Black, and other races—including multi-racial), BMI [underweight (<18.5 kg/m^2^), normal weight (≥18.5 and <25 kg/m^2^), overweight (≥25 and <30 kg/m^2^), class I obese (≥30 and <35 kg/m^2^), class II obese (≥35 and <40 kg/m^2^), class III obese (≥40 kg/m^2^), and missing values], year of collecting data, and physical activity (MET-hrs./week = 0, quartiles, missing). ^2^Diets were classified based on macronutrient energy distribution. A balanced diet was defined as 10–35% protein, 45–65% carbohydrate, and 20–35% fat. Low-protein: protein <10%, carbohydrate 45–65%, fat 20–35%. Low-protein, high-fat: protein <10%, carbohydrate 45–65%, fat >35%. High-fat: protein 10–35%, carbohydrate 45–65%, fat >35%. Unbalanced (all off-range): protein <10%, carbohydrate <45%, fat >35%. Low-carbohydrate, high-fat: protein 10–35%, carbohydrate <45%, fat >35%. Low-carbohydrate: protein 10–35%, carbohydrate <45%, fat 20–35%.

**Figure 4:**
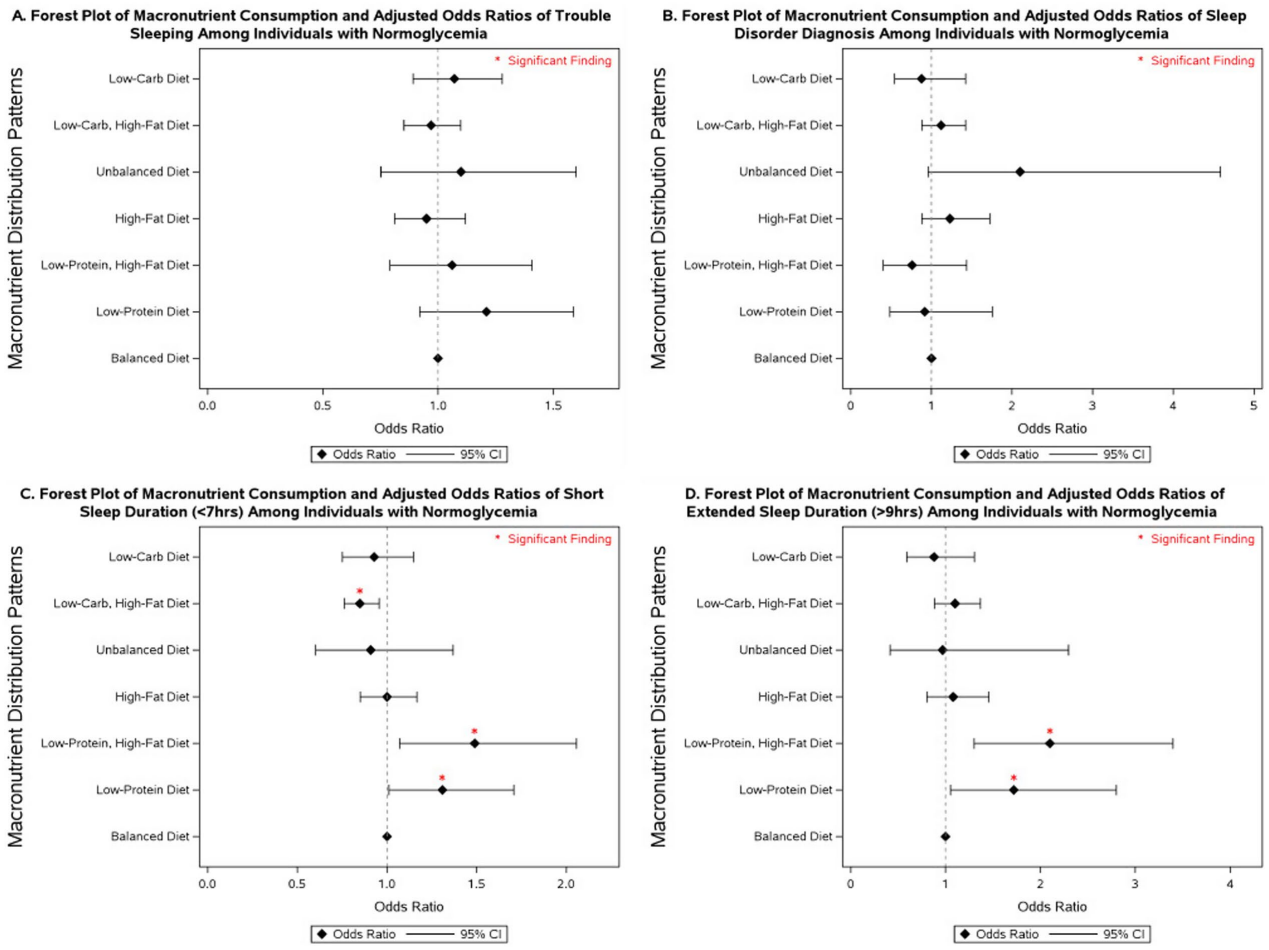
Forest plot of fully adjusted^1^ odds ratios for the associations between macronutrient intake^2^ and sleep outcomes among participants with normoglycemia in NHANES 2007–2020 (*n* = 17,908). Black diamonds represent odds ratios (ORs), with horizontal lines indicating 95% confidence intervals (CIs); statistically significant findings are marked with a red asterisk. ^1^The model adjusts for sex (men and women), age (18-34, 35-44, 45-54, 55–64, and ≥65 years), race/ethnicity (Non-Hispanic White, Mexican American, Non-Mexican Hispanic, Non-Hispanic Black, and other races including multi-racial), BMI [underweight (<18.5 kg/m^2^), normal weight (≥18.5 and <25 kg/m^2^), overweight (≥25 and <30 kg/m^2^), class I obese (≥30 and <35 kg/m^2^), class II obese (≥35 and <40 kg/m^2^), class III obese (≥40 kg/m^2^), and missing values], year of collecting data, and physical activity (MET-hrs./week = 0, quartiles, missing). ^2^ Diets were classified based on macronutrient energy distribution. A balanced diet was defined as 10–35% protein, 45–65% carbohydrate, and 20–35% fat. Low-protein: protein <10%, carbohydrate 45–65%, fat 20–35%. Low-protein, high-fat: protein <10%, carbohydrate 45–65%, fat >35%. High-fat: protein 10–35%, carbohydrate 45–65%, fat >35%. Unbalanced (all off-range): protein <10%, carbohydrate <45%, fat >35%. Low-carbohydrate, high-fat: protein 10–35%, carbohydrate <45%, fat >35%. Low-carbohydrate: protein 10–35%, carbohydrate <45%, fat 20–35%.

More detailed results on the associations between macronutrient energy distribution patterns and sleep outcomes across glycemic status groups are presented in Figures 2–4 and Supplementary Tables 4–6.

## 4 Discussion

Overall, individuals with diabetes had significantly higher odds of reporting trouble sleeping, being diagnosed with sleep disorders, and experiencing abnormal sleep durations, including both short and extended sleep, compared to individuals with normoglycemia. Although prediabetes was associated only with higher odds of trouble sleeping in the fully adjusted model, when adjusting solely for non-modifiable covariates, it was also associated with diagnosed sleep disorders and shorter sleep duration. These results align with previous NHANES studies showing that individuals with diabetes were more likely to have short sleep duration (50) and that poor sleep quality was associated with higher diabetes prevalence (58). Other studies have also linked both short (≤6 h) and long (≥9 h) sleep with increased prevalence of type 2 diabetes (59) and found that sleeping less than or equal to 5 h doubled the odds of prediabetes compared to normal sleep duration (60). To our knowledge, this was the first study examining the association between diabetes control status and sleep outcomes in this population. Among individuals with diabetes, maintaining HbA₁c levels below 6.5% was linked to significantly higher odds of trouble sleeping compared to maintaining levels that are ≥ 6.5% and < 9.0%. Our previous analysis of NHANES data showed that strict HbA₁c control (HbA₁c < 6.5) was associated with an increased risk of depression in this population (53), which may, in turn, impact their sleep. Another potential explanation, beyond psychological factors such as depression, is that tighter glycemic control in diabetes often requires more intensive treatment regimens, which can increase the risk of nocturnal hypoglycemia, a well-documented disruptor of sleep quality (61–64). In addition, individuals under stricter treatment protocols may experience a greater disease burden or medication-related side effects, particularly from insulin or sulfonylureas, which are known to contribute to sleep difficulties (65, 66). Diabetes medications, especially long-term metformin, can also affect nutritional status by impairing vitamin B₁₂ absorption, primarily through interference with calcium-dependent binding of the intrinsic factor–B₁₂ complex in the ileum (67, 68). Reduced serum B₁₂ levels have been associated with poor sleep quality and increased daytime sleepiness (69). These mechanisms warrant further investigation to clarify whether the observed association reflects treatment effects, comorbidity burden, or other behavioral/psychological factors. Overall, these findings suggest that glycemic status and diabetes control are closely linked to both sleep quantity and quality, highlighting a potentially bidirectional relationship. Future studies should examine the interrelationship between diabetes control and sleep outcomes and how this might influence diabetes management and the overall well-being of patients with diabetes.

Macronutrient energy distribution was also associated with sleep outcomes, and these associations varied by glycemic status. In individuals with diabetes, low-protein diets were linked to increased odds of disordered sleep, whereas low-carbohydrate, high-fat diets were associated with reduced odds of short sleep duration. Among those with prediabetes, low-protein diets, especially when combined with high-fat intake, were associated with a 2- to 3-fold higher likelihood of extended sleep duration. In normoglycemic individuals, low-protein intake was linked to both short and extended sleep duration, with stronger associations observed when combined with high-fat intake. By contrast, low-carbohydrate, high-fat diets in this group were associated with lower odds of short sleep duration. These findings underscore the importance of considering both glycemic status and dietary macronutrient composition in strategies aimed at improving sleep health. Our results align with a study that reported an inverse relationship between sleep stages and macronutrient intake, noting that higher fat consumption was associated with sleep disturbances, particularly among individuals with obesity (37). Another study using NHANES data from 2005 to 2014 found that a high intake of solid fats was associated with an increased risk of sleep disorders and shorter sleep duration (70). A systematic review concluded that higher dietary protein intake may improve sleep quality among healthy individuals (32). Consistently, Lindseth et al. reported that participants experienced fewer nocturnal awakenings when consuming high-protein diets (71), and a randomized clinical trial demonstrated that global sleep scores significantly improved in the high-protein group but not in the normal-protein group after 16 weeks of follow-up (40). These benefits are often attributed to tryptophan, an essential amino acid abundant in protein, which serves as a precursor for serotonin and melatonin synthesis, thereby influencing sleep regulation (24, 26, 72, 73). Collectively, this evidence underscores the role of dietary protein in promoting sleep quality through both behavioral and biochemical pathways. An analysis of NHANES data (2005–2008 and 2015-2018) by Zhao et al. reported that higher carbohydrate intake was associated with increased odds of sleepiness and snoring but found no significant link to abnormal sleep duration in adjusted models (39). We observed no significant association between low-carbohydrate diets and abnormal sleep outcomes in our fully adjusted models. However, in individuals with diabetes, low-carbohydrate diets were associated with lower odds of extended sleep duration when models were adjusted only for non-modifiable covariates. Our findings also indicated that low-carbohydrate intake when combined with high-fat diets was associated with a reduction in the odds of short sleep duration in individuals with diabetes and normoglycemia compared to balanced diets.

A major strength of this analysis is the use of nationally representative NHANES data collected over 14 years, enhancing the generalizability of our findings. Additionally, to our knowledge, this is the first study to examine the associations between macronutrient distribution patterns, defined according to AMDR recommendations, and sleep outcomes across three distinct glycemic statuses. This is particularly significant, as our findings indicate that macronutrient-sleep associations differ depending on glycemic status. Comprehensive adjustment for demographic and lifestyle factors further strengthens the validity of our results. Thus, this study offers critical initial insights that provide groundwork for future clinical trials aimed at establishing dietary recommendations to improve sleep outcomes. This study also has several limitations that should be considered. The cross-sectional nature of the study restricts our ability to establish temporal relationships. Additionally, reliance on self-reported dietary intake may have influenced our findings, potentially introducing misclassification bias; however, this limitation is partially mitigated by incorporating reliability ratings assigned by NHANES examiners. Dietary intake was assessed using a single 24-h dietary recall. Sleep patterns are more likely influenced by habitual dietary intake over time rather than intake from 1 day, and thus, our results may not fully capture usual dietary behaviors. Future studies should incorporate repeated dietary assessments to better reflect long-term dietary patterns in relation to sleep outcomes. Some associations between macronutrient distribution patterns and sleep outcomes were accompanied by wide confidence intervals, likely reflecting smaller subgroup sizes. In particular, findings for low-protein diets among participants with diabetes and prediabetes should be interpreted with caution. By contrast, estimates for the normoglycemia group, as well as for low-carbohydrate, high-fat patterns in both diabetes and normoglycemia, were comparatively more precise. Key confounders such as depression, anxiety, and medication use (e.g., insulin, sedatives, antidepressants) were not accounted for in this study, which may have influenced both glycemic control and sleep outcomes.

In summary, our findings indicate that individuals with diabetes have a significantly higher risk of impaired sleep quality and abnormal sleep duration, including both short and extended sleep, compared to those without diabetes, with a similar but less pronounced pattern observed in individuals with prediabetes. These results underscore the need for increased screening and management of sleep disorders in both diabetic and prediabetic populations to enhance overall quality of life. Additionally, macronutrient energy distribution is linked to sleep outcomes, with associations varying by glycemic status. Low-protein and low-protein, high-fat diets were most consistently related to poor sleep, highlighting the need to consider both diet and glycemic status in sleep health strategies. Future studies should explore the causal relationships between macronutrient energy distribution and sleep outcomes using longitudinal or intervention-based designs, while also accounting for glycemic status to better inform targeted dietary recommendations.

## Data Availability

Publicly available datasets were analyzed in this study. This data can be found on the CDC website: https://wwwn.cdc.gov/nchs/nhanes/.
