# Supplementary material for: Glycemic status and macronutrient intake as predictors of sleep outcomes: an analysis of NHANES 2007–2020 data
Source: Front Nutr. 2025 Oct 20;12:1672631. doi: 10.3389/fnut.2025.1672631 (PMC12580088; doi:10.3389/fnut.2025.1672631)
Supplement: Supplementary file 1 [file Data_Sheet_1.pdf]

Supplementary Material

1 Supplementary Figures and Tables

Supplementary Table 1. Dietary Characteristics of NHANES Participants Eligible for the Dietary Analysis Portions of the Study (2007–2020), Stratified by Glycemic Status

	**Total Participants (n = 29,964)**	**Participants with Normoglycemia (n = 17,908)**	**Participants with Prediabetes (n = 7,181)**	**Participants with Diabetes (n = 4,875)**
**Macronutrient Balance Consumption Pattern**				
Balanced Diet[1]	31.38% (n=10,139)	31.87% (n=6,117)	32.08% (n=2,496)	27.56% (n=1,526)
Low-Protein Diet[2]	2.87% (n=893)	3.08% (n=587)	2.90% (n=225)	1.69% (n=81)
Low-Protein, High-Fat Diet[3]	2.32% (n=815)	2.28% (n=498)	2.51% (n=205)	2.27% (n=112)
High-Fat Diet[4]	13.33% (n= 4,123)	12.93% (n=2,396)	14.77% (n=1,078)	13.12% (n=649)
Unbalanced Diet[5]	1.20% (n=333)	1.30% (n=214)	1.03% (n=76)	0.96% (n=43)
Low-Protein, Low-Carb Diet[6]	0.60% (n=183)	0.65% (n=123)	0.59% (n=43)	0.33% (n=17)
Low-Carb, High-Fat Diet[7]	38.65% (n=10,765)	37.19% (n=6,110)	38.75% (n=2,546)	46.46% (n=2,109)
Low-Carb Diet[8]	9.64% (n=2,713)	10.71% (n=1,863)	7.38% (n=512)	7.61% (n=338)

Proportions (%) are weighted using NHANES sampling weights to represent the non-institutionalized resident US population. Counts (n) are unweighted and represent the number of study participants within each subgroup.
[1]Balanced Diet: 10% ≤ Protein ≤ 35%, 45% ≤ Carbohydrate ≤ 65%, 20% ≤ Fat ≤ 35%
[2]Low-Protein: Protein <10%, 45% ≤ Carbohydrate ≤ 65%, 20% ≤ Fat ≤ 35%
[3]Low-Protein, High-Fat: Protein <10%, 45% ≤ Carbohydrate ≤ 65%, Fat >35%
[4]High-Fat: 10% ≤ Protein ≤ 35%, 45% ≤ Carbohydrate ≤ 65%, Fat >35%
[5]Unbalanced (All Off-Range): Protein <10%, Carbohydrate <45%, Fat >35%

[6]Low-Protein, Low-Carb: Protein <10%, Carbohydrate <45%, 20% ≤ Fat ≤ 35%<br>[7]Low-Carb, High-Fat: 10% ≤ Protein ≤ 35%, Carbohydrate <45%, Fat >35%<br>[8]Low-Carb: 10% ≤ Protein ≤ 35%, Carbohydrate <45%, 20% ≤ Fat ≤ 35%

Supplementary Table 2. Unadjusted and Semi-adjusted Odds Ratios and 95% Confidence Intervals for Associations between Glycemic Status and Sleep Outcomes among NHANES Participants (n = 39,794)

	**Unadjusted Model[1]**			**Semi-Adjusted Model[2]**		
	**OR**	**Lower Limit**	**Upper Limit**	**OR**	**Lower Limit**	**Upper Limit**
**Sleep Quality**						
Trouble Sleeping						
Normoglycemia	1.00	Ref	Ref	1.00	Ref	Ref
Prediabetes	1.35	1.25	1.46	1.19	1.09	1.29
Diabetes	1.83	1.66	2.01	1.64	1.47	1.83
Sleep Disorder Diagnosis						
Normoglycemia	1.00	Ref	Ref	1.00	Ref	Ref
Prediabetes	1.60	1.36	1.88	1.42	1.19	1.68
Diabetes	3.03	2.59	3.55	2.68	2.22	3.24
**Sleep Quantity**						
Short (<7hrs) Duration						
Normoglycemia	1.00	Ref	Ref	1.00	Ref	Ref
Prediabetes	1.09	1.02	1.17	1.08	1.01	1.16
Diabetes	1.27	1.15	1.41	1.30	1.16	1.45
Extended (>9hrs) Duration						
Normoglycemia	1.00	Ref	Ref	1.00	Ref	Ref
Prediabetes	1.05	0.92	1.20	0.99	0.87	1.14
Diabetes	1.67	1.40	1.99	1.51	1.27	1.81
[1]Depicts the results of an unadjusted model<br>[2]Depicts the results of a partially-adjusted model, adjusting for sex (men and women), age (18–34, 35–44, 45–54, 55–64, and ≥65						

years), race/ethnicity (Non-Hispanic White, Mexican American, Non-Mexican Hispanic, Non-Hispanic Black, and Other Races—Including Multi-Racial).

Supplementary Table 3. Unadjusted and Semi-adjusted Odds Ratios and 95% Confidence Intervals for Associations between Diabetes Control Status and Sleep Outcomes among Participants with Diabetes in NHANES (n = 5,855)

	**Unadjusted Model[1]**			**Semi-Adjusted Model[2]**		
	**OR**	**Lower Limit**	**Upper Limit**	**OR**	**Lower Limit**	**Upper Limit**
**Sleep Quality**						
Trouble Sleeping						
Diabetes ($HbA_{1c} \geq 9$)	0.95	0.76	1.18	0.97	0.77	1.22
Diabetes ($6.5 \leq HbA_{1c} < 9$)	1.00	Ref	Ref	1.00	Ref	Ref
Diabetes ($HbA_{1c} < 6.5$)	1.25	1.04	1.51	1.21	1.00	1.45
Sleep Disorder Diagnosis						
Diabetes ($HbA_{1c} \geq 9$)	0.96	0.64	1.44	0.98	0.66	1.48
Diabetes ($6.5 \leq HbA_{1c} < 9$)	1.00	Ref	Ref	1.00	Ref	Ref
Diabetes ($HbA_{1c} < 6.5$)	1.13	0.78	1.62	1.15	0.79	1.65
**Sleep Quantity**						
Short (<7hrs) Duration						
Diabetes ($HbA_{1c} \geq 9$)	1.35	1.03	1.77	1.22	0.92	1.62
Diabetes ($6.5 \leq HbA_{1c} < 9$)	1.00	Ref	Ref	1.00	Ref	Ref
Diabetes ($HbA_{1c} < 6.5$)	0.99	0.84	1.18	0.99	0.83	1.18
Extended (>9hrs) Duration						
Diabetes ($HbA_{1c} \geq 9$)	0.79	0.53	1.17	0.85	0.56	1.27
Diabetes ($6.5 \leq HbA_{1c} < 9$)	1.00	Ref	Ref	1.00	Ref	Ref
Diabetes ($HbA_{1c} < 6.5$)	1.01	0.73	1.40	0.99	0.71	1.36

[1]Depicts the results of an unadjusted model
[2]Depicts the results of a partially-adjusted model, adjusting for sex (men and women), age (18–34, 35–44, 45–54, 55–64, and ≥65 years), race/ethnicity (Non-Hispanic White, Mexican American, Non-Mexican Hispanic, Non-Hispanic Black, and Other Races—Including Multi-Racial).

Supplementary Table 4. Fully-Adjusted Odds Ratios for the Associations between Macronutrient Intake and Sleep Outcomes among Participants with Diabetes, Prediabetes, and Normoglycemia in NHANES 2007–2020 (n=29,964)

	**Participants with Normoglycemia (n = 17,908)**			**Participants with Prediabetes (n = 7,181)**			**Participants with Diabetes (n = 4,875)**		
	**OR**	**Lower Limit**	**Upper Limit**	**OR**	**Lower Limit**	**Upper Limit**	**OR**	**Lower Limit**	**Upper Limit**
Sleep Quality									
Trouble Sleeping									
Balanced Diet[1]	1.00	Ref	Ref	1.00	Ref	Ref	1.00	Ref	Ref
Low-Protein Diet[2]	1.21	0.92	1.59	0.87	0.55	1.39	1.21	0.70	2.09
Low-Protein, High-Fat Diet[3]	1.06	0.79	1.41	0.98	0.62	1.54	0.90	0.58	1.41
High-Fat Diet[4]	0.95	0.81	1.12	0.99	0.79	1.25	1.27	0.96	1.68
Unbalanced Diet[5]	1.10	0.75	1.60	1.15	0.63	2.11	0.63	0.27	1.44
Low-Carb, High-Fat Diet [6]	0.97	0.85	1.10	1.05	0.86	1.28	1.01	0.80	1.26
Low-Carb Diet[7]	1.07	0.89	1.28	0.97	0.70	1.35	0.67	0.44	1.03
Sleep Disorder Diagnosis									
Balanced Diet[1]	1.00	Ref	Ref	1.00	Ref	Ref	1.00	Ref	Ref
Low-Protein Diet[2]	0.92	0.48	1.76	0.56	0.29	1.06	2.43	1.06	5.61
Low-Protein, High-Fat Diet[3]	0.76	0.40	1.44	0.77	0.36	1.67	0.94	0.33	2.67
High-Fat Diet[4]	1.23	0.88	1.73	0.74	0.48	1.15	1.31	0.87	1.97
Unbalanced Diet[5]	2.10	0.96	4.59	1.30	0.34	5.04	2.02	0.56	7.31
Low-Carb, High-Fat Diet[6]	1.12	0.88	1.43	0.87	0.63	1.20	1.08	0.76	1.54
Low-Carb Diet[7]	0.88	0.54	1.43	0.97	0.61	1.55	0.61	0.35	1.07
Sleep Quantity									

Short Sleep Duration (<7hrs)									
Balanced Diet[1]	1.00	Ref	Ref	1.00	Ref	Ref	1.00	Ref	Ref
Low-Protein Diet[2]	1.31	1.01	1.71	1.14	0.70	1.87	0.66	0.32	1.39
Low-Protein, High-Fat Diet[3]	1.49	1.07	2.06	1.33	0.90	1.96	0.89	0.55	1.43
High-Fat Diet[4]	1.00	0.85	1.17	1.12	0.86	1.46	1.07	0.80	1.43
Unbalanced Diet[5]	0.91	0.60	1.37	0.71	0.42	1.19	0.74	0.30	1.84
Low-Carb, High-Fat Diet[6]	0.85	0.76	0.96	1.09	0.89	1.34	0.78	0.62	0.98
Low-Carb Diet[7]	0.93	0.75	1.15	0.84	0.57	1.24	1.01	0.69	1.48
Extended Sleep Duration (>9hrs)									
Balanced Diet[1]	1.00	Ref	Ref	1.00	Ref	Ref	1.00	Ref	Ref
Low-Protein Diet[2]	1.72	1.05	2.80	2.04	1.02	4.08	0.84	0.32	2.18
Low-Protein, High-Fat Diet[3]	2.10	1.30	3.40	2.88	1.30	6.36	1.39	0.58	3.38
High-Fat Diet[4]	1.08	0.80	1.46	1.61	0.92	2.82	0.97	0.51	1.86
Unbalanced Diet[5]	0.97	0.41	2.30	0.68	0.23	2.04	1.24	0.38	4.04
Low-Carb, High-Fat Diet[6]	1.10	0.88	1.37	0.80	0.57	1.11	0.94	0.65	1.36
Low-Carb Diet[7]	0.88	0.59	1.31	0.71	0.34	1.46	0.49	0.24	1.02

Model adjusts for sex (men and women), age (18–34, 35–44, 45–54, 55–64, and ≥65 years), race/ethnicity (Non-Hispanic White, Mexican American, Non-Mexican Hispanic, Non-Hispanic Black, and other races—including multi-racial), BMI [underweight (<18.5 kg/m2), normal weight (≥18.5 and <25 kg/m2), overweight (≥25 and <30 kg/m2), class I obese (≥30 and <35 kg/m2), class II obese (≥35 and <40 kg/m2), class III obese (≥40 kg/m2), and missing values], year of collecting data (continuous), and physical activity (MET-hrs./week = 0, quartiles, missing).

[1]Balanced Diet: 10% ≤ Protein ≤ 35%, 45% ≤ Carbohydrate ≤ 65%, 20% ≤ Fat ≤ 35%

[2]Low-Protein: Protein <10%, 45% ≤ Carbohydrate ≤ 65%, 20% ≤ Fat ≤ 35%

[3]Low-Protein, High-Fat: Protein <10%, 45% ≤ Carbohydrate ≤ 65%, Fat >35%

[4]High-Fat: 10% ≤ Protein ≤ 35%, 45% ≤ Carbohydrate ≤ 65%, Fat >35%

[5]Unbalanced (All Off-Range): Protein <10%, Carbs <45%, Fat >35%

[6]Low-Carb, High-Fat: 10% ≤ Protein ≤ 35%, Carbohydrate <45%, Fat >35%

[7]Low-Carb: 10% ≤ Protein ≤ 35%, Carbohydrate <45%, 20% ≤ Fat ≤ 35%

Supplementary Table 5. Semi-Adjusted Odds Ratios for the Associations between Macronutrient Intake and Sleep Outcomes among Participants with Diabetes, Prediabetes, and Normoglycemia in NHANES 2007–2020 (n=29,964)

	**Participants with Normoglycemia (n = 17,908)**			**Participants with Prediabetes (n = 7,181)**			**Participants with Diabetes (n = 4,875)**		
	**OR**	**Lower Limit**	**Upper Limit**	**OR**	**Lower Limit**	**Upper Limit**	**OR**	**Lower Limit**	**Upper Limit**
Sleep Quality									
Trouble Sleeping									
Balanced Diet[1]	1.00	Ref	Ref	1.00	Ref	Ref	1.00	Ref	Ref
Low-Protein Diet[2]	1.22	0.93	1.60	0.92	0.57	1.49	1.23	0.68	2.22
Low-Protein, High-Fat Diet[3]	1.09	0.83	1.42	0.99	0.63	1.54	0.89	0.56	1.40
High-Fat Diet[4]	0.98	0.84	1.15	1.02	0.81	1.28	1.28	0.97	1.69
Unbalanced Diet[5]	1.09	0.75	1.59	1.21	0.65	2.25	0.63	0.28	1.44
Low-Carb, High-Fat Diet[6]	1.00	0.88	1.13	1.10	0.90	1.34	1.04	0.84	1.28
Low-Carb Diet[7]	1.07	0.89	1.28	0.98	0.71	1.34	0.68	0.45	1.02
Sleep Disorder Diagnosis									
Balanced Diet[1]	1.00	Ref	Ref	1.00	Ref	Ref	1.00	Ref	Ref
Low-Protein Diet[2]	0.89	0.48	1.65	0.60	0.33	1.11	2.71	1.15	6.39
Low-Protein, High-Fat Diet[3]	0.80	0.41	1.55	0.77	0.37	1.63	0.85	0.30	2.36
High-Fat Diet[4]	1.27	0.91	1.76	0.85	0.55	1.33	1.38	0.94	2.03
Unbalanced Diet[5]	1.89	0.82	4.32	1.25	0.32	4.85	2.23	0.61	8.09
Low-Carb, High-Fat Diet[6]	1.17	0.92	1.48	1.02	0.74	1.41	1.10	0.80	1.50
Low-Carb Diet[7]	0.86	0.53	1.39	0.99	0.62	1.56	0.61	0.34	1.08
Sleep Quantity									
Short Sleep Duration (<7hrs)									
Balanced Diet[1]	1.00	Ref	Ref	1.00	Ref	Ref	1.00	Ref	Ref
Low-Protein Diet[2]	1.30	0.98	1.71	1.14	0.71	1.84	0.64	0.32	1.30
Low-Protein, High-Fat Diet[3]	1.42	1.02	1.97	1.27	0.85	1.88	0.80	0.50	1.28

High-Fat Diet[4]	1.00	0.86	1.17	1.08	0.83	1.41	1.07	0.80	1.44
Unbalanced Diet[5]	0.88	0.58	1.34	0.65	0.38	1.12	0.74	0.30	1.78
Low-Carb, High-Fat Diet[6]	0.82	0.73	0.93	1.04	0.85	1.28	0.76	0.61	0.94
Low-Carb Diet[7]	0.91	0.74	1.12	0.83	0.56	1.22	1.00	0.68	1.46
Extended Sleep Duration (>9hrs)									
Balanced Diet[1]	1.00	Ref	Ref	1.00	Ref	Ref	1.00	Ref	Ref
Low-Protein Diet[2]	1.83	1.14	2.93	2.42	1.14	5.12	0.99	0.38	2.54
Low-Protein, High-Fat Diet[3]	2.39	1.46	3.91	2.93	1.35	6.35	1.48	0.62	3.57
High-Fat Diet[4]	1.21	0.89	1.63	1.77	1.02	3.06	1.00	0.54	1.85
Unbalanced Diet[5]	1.18	0.53	2.65	0.82	0.27	2.47	1.51	0.44	5.16
Low-Carb, High-Fat Diet[6]	1.22	0.97	1.52	0.87	0.62	1.21	0.97	0.68	1.38
Low-Carb Diet[7]	0.88	0.59	1.31	0.72	0.36	1.44	0.48	0.24	0.96

Model adjusts for sex (men and women), age (18–34, 35–44, 45–54, 55–64, and ≥65 years), race/ethnicity (Non-Hispanic White, Mexican American, Non-Mexican Hispanic, Non-Hispanic Black, and other races—including multi-racial).
[1]Balanced Diet: 10% ≤ Protein ≤ 35%, 45% ≤ Carbohydrate ≤ 65%, 20% ≤ Fat ≤ 35%
[2]Low-Protein: Protein <10%, 45% ≤ Carbohydrate ≤ 65%, 20% ≤ Fat ≤ 35%
[3]Low-Protein, High-Fat: Protein <10%, 45% ≤ Carbohydrate ≤ 65%, Fat >35%
[4]High-Fat: 10% ≤ Protein ≤ 35%, 45% ≤ Carbohydrate ≤ 65%, Fat >35%
[5]Unbalanced (All Off-Range): Protein <10%, Carbs <45%, Fat >35%
[6]Low-Carb, High-Fat: 10% ≤ Protein ≤ 35%, Carbohydrate <45%, Fat >35%
[7]Low-Carb: 10% ≤ Protein ≤ 35%, Carbohydrate <45%, 20% ≤ Fat ≤ 35%

Supplementary Table 6. Unadjusted Odds Ratios for the Associations between Macronutrient Intake and Sleep Outcomes among Participants with Diabetes, Prediabetes, and Normoglycemia in NHANES 2007–2020 (n=29,964)

	Participants with Normoglycemia (n = 17,908)	Participants with Prediabetes (n = 7,181)	Participants with Diabetes (n = 4,875)

	OR	Lower Limit	Upper Limit	OR	Lower Limit	Upper Limit	OR	Lower Limit	Upper Limit
Sleep Quality									
Trouble Sleeping									
Balanced Diet[1]	1.00	Ref	Ref	1.00	Ref	Ref	1.00	Ref	Ref
Low-Protein Diet[2]	1.24	0.96	1.61	0.94	0.59	1.50	1.29	0.69	2.41
Low-Protein, High-Fat Diet[3]	1.12	0.85	1.46	1.10	0.71	1.70	0.88	0.56	1.39
High-Fat Diet[4]	0.99	0.85	1.16	1.08	0.86	1.34	1.31	0.99	1.74
Unbalanced Diet[5]	1.22	0.83	1.79	1.18	0.64	2.18	0.61	0.26	1.42
Low-Carb, High-Fat Diet[6]	1.05	0.92	1.19	1.15	0.95	1.41	1.05	0.85	1.30
Low-Carb Diet[7]	1.11	0.92	1.33	1.01	0.74	1.37	0.67	0.45	1.00
Sleep Disorder Diagnosis									
Balanced Diet[1]	1.00	Ref	Ref	1.00	Ref	Ref	1.00	Ref	Ref
Low-Protein Diet[2]	0.93	0.51	1.70	0.59	0.33	1.07	2.79	1.13	6.93
Low-Protein, High-Fat Diet[3]	0.76	0.39	1.48	0.83	0.40	1.73	0.84	0.30	2.35
High-Fat Diet[4]	1.29	0.93	1.80	0.88	0.57	1.36	1.45	0.99	2.13
Unbalanced Diet[5]	2.17	1.00	4.74	1.09	0.29	4.17	2.15	0.56	8.29
Low-Carb, High-Fat Diet[6]	1.26	0.99	1.61	1.08	0.79	1.48	1.23	0.89	1.68
Low-Carb Diet[7]	0.95	0.59	1.52	1.05	0.67	1.65	0.71	0.41	1.23
Sleep Quantity									
Short Sleep Duration (<7hrs)									
Balanced Diet[1]	1.00	Ref	Ref	1.00	Ref	Ref	1.00	Ref	Ref
Low-Protein Diet[2]	1.33	1.01	1.75	1.14	0.71	1.82	0.68	0.34	1.39
Low-Protein, High-Fat Diet[3]	1.43	1.03	1.98	1.30	0.88	1.92	0.79	0.51	1.22
High-Fat Diet[4]	1.02	0.88	1.18	1.04	0.80	1.34	1.07	0.81	1.42
Unbalanced Diet[5]	0.84	0.56	1.26	0.68	0.40	1.15	0.68	0.29	1.60
Low-Carb, High-Fat Diet[6]	0.83	0.73	0.93	1.03	0.85	1.24	0.75	0.61	0.92
Low-Carb Diet[7]	0.94	0.76	1.14	0.85	0.58	1.24	0.98	0.67	1.41
Extended Sleep Duration (>9hrs)									
Balanced Diet[1]	1.00	Ref	Ref	1.00	Ref	Ref	1.00	Ref	Ref

Low-Protein Diet[2]	1.79	1.11	2.89	2.45	1.18	5.06	0.95	0.37	2.47
Low-Protein, High-Fat Diet[3]	2.59	1.59	4.24	3.09	1.43	6.66	1.59	0.66	3.85
High-Fat Diet[4]	1.22	0.90	1.65	1.79	1.04	3.08	0.99	0.54	1.82
Unbalanced Diet[5]	1.19	0.54	2.63	0.88	0.29	2.64	1.60	0.48	5.38
Low-Carb, High-Fat Diet[6]	1.14	0.91	1.42	0.84	0.60	1.18	0.92	0.64	1.30
Low-Carb Diet[7]	0.78	0.52	1.15	0.66	0.34	1.29	0.43	0.22	0.86

[1]Balanced Diet: 10% ≤ Protein ≤ 35%, 45% ≤ Carbohydrate ≤ 65%, 20% ≤ Fat ≤ 35%
[2]Low-Protein: Protein <10%, 45% ≤ Carbohydrate ≤ 65%, 20% ≤ Fat ≤ 35%
[3]Low-Protein, High-Fat: Protein <10%, 45% ≤ Carbohydrate ≤ 65%, Fat >35%
[4]High-Fat: 10% ≤ Protein ≤ 35%, 45% ≤ Carbohydrate ≤ 65%, Fat >35%
[5]Unbalanced (All Off-Range): Protein <10%, Carbs <45%, Fat >35%
[6]Low-Carb, High-Fat: 10% ≤ Protein ≤ 35%, Carbohydrate <45%, Fat >35%
[7]Low-Carb: 10% ≤ Protein ≤ 35%, Carbohydrate <45%, 20% ≤ Fat ≤ 35%
